# Radical Electroprecipitation from a Water|Oil|Electrode Interface Prolongs “Electro”Chemiluminescence of the Tris(2,2′‐bipyridyl)Ruthenium(II) and Benzoyl Peroxide System by 10^3^

**DOI:** 10.1002/anie.202521454

**Published:** 2026-01-07

**Authors:** Daniel M. Carrel, Brady R. Layman, Megan L. Hill, Jeffrey E. Dick

**Affiliations:** ^1^ Department of Chemistry Purdue University West Lafayette IN 47907 USA

**Keywords:** Electrochemiluminescence, ECL Microscopy, Interface, Radical

## Abstract

Reactive intermediates, including radicals and other short‐lived molecules, are ubiquitous in pure chemical processes and often highly difficult to isolate. Recently, we found that energetic, reactive radical species can be trapped through electroprecipitation, where the precipitate contains a reactive intermediate. The principle is straight‐forward: If a solubility equilibrium can be exceeded before the reactive intermediate lifetime, the intermediate can be precipitated. Classically, electrochemiluminescence (ECL) is a process where electrical energy is transferred to chemical energy, and then the chemical energy is released in the form of light emission (often in the presence of an electroactive luminophore). The apparent luminescence lifetime, limited by radical lifetimes, of ECL reactions in solution is short (sub‐microsecond), and thus can hinder the performance of the technique as light emission is confined to the electrode surface and occurs only when the electrical potential to the system is applied. Here, we show that with a high concentration (10–250 mM) of benzoyl peroxide (BPO) being co‐reduced with a tris(2,2′‐bipyridyl)ruthenium(II) complex, we can achieve an ECL emission that lasts for hundreds of seconds after potential arrest, corresponding to a thousand‐fold (10^3^) increase in apparent afterglow chemiluminescence lifetimes. This electroprecipitation process occurs at an aqueous|organic multiphase boundary, which are present all through nature and are arguably more representative of natural chemical processes. Furthermore, experimental parameters, including BPO concentration, applied potential, and electrode material, were investigated. Competitive and parasitic processes, such as the hydrogen evolution reaction and decarboxylation, diminish the electroprecipitation of reactive intermediates, and we detail mitigation and optimization efforts. Overall, this work reveals that multiphase interfaces can be used to electroprecipitate reactive radicals, separating their use in space and time.

## Graphical abstract

Multiphase systems, including ones with liquid‐liquid phase boundaries, are the home to many curious chemistries including reaction acceleration and spontaneous formation of reactive species. Here we show the liquid‐liquid interface causes electrochemiluminescence and afterglow chemiluminescence lifetime to be enhanced by 220,000% in‐time, far beyond when the electrical potential is applied.

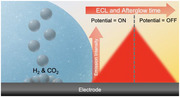

## Introduction

Radicals and reactive intermediates pervade nature and are implicated in important chemical mechanisms throughout pure chemistry.^[^ ^1^, ^2^, ^3^, ^4^ ^]^ Most of these radicals are elusive, with very short lifetimes (nano‐ to microsecond), and, as a result, only a handful are commercially available. Developing methods to extend radical lifetimes can have powerful synthetic applications. Furthermore, as heterogeneous electrochemical reactions often proceed with only one electron at a time^[^ ^5^ ^]^ electroanalytical chemistry is an ideal candidate for the study of these elusive species.^[^ ^6^ ^]^ Notably, with electroanalytical chemistry, one can selectively generate a controllable concentration gradient that produces these radicals for use within an investigation.^[^ ^6^ ^]^

Electrochemistry is an inherently multiphase (liquid|electrode) field that has been widely used to explore interfacial reactivity, namely liquid|liquid, liquid|solid, and liquid|air, which are prevalent throughout multiphase environments inherent in energy storage and conversion, biosensors, and photoelectrochemistry.^[^ ^7^, ^8^, ^9^ ^]^ We previously demonstrated the ability to heterogeneously electroprecipitate the sulfate radical anion.^[^ ^10^ ^]^ We took advantage of the solubility product equilibrium: If the anion could be generated at a high enough concentration to exceed solubility within its radical lifetime, the radical could be fossilized and used for future reactions. Electrochemistry is helpful because locally high concentration profiles can be driven that are not achievable under homogeneous conditions.^[^ ^11^ ^]^

In our prior work, we applied such radical entrapment to electrochemiluminescence (ECL).^[^ ^10^ ^]^ ECL is a light‐emitting technique, where electrogenerated intermediates react with an electroactive luminophore, evolving light.^[^ ^12^, ^13^ ^]^ In general, a luminophore is either oxidized or reduced in the presence of a coreactant, which is oxidized or reduced and undergoes a chemical decomposition to create a strong reducing agent or oxidizing agent, respectively.^[^ ^14^ ^]^ Our group has also been interested in multiphase reactivity as it has departed from conventional pure chemistry studies in the bulk, continuous phase,^[^ ^15^, ^16^, ^17^ ^]^ and we have spent considerable effort using ECL microscopy to understand physicochemical properties of multiphase environments.^[^ ^18^, ^19^, ^20^, ^21^, ^22^ ^]^ Other *in operando* studies have shown that catalytic and electron‐transfer rates of surface‐immobilized catalysis systems are of the same magnitude, emphasizing the importance of physical mass and electron transport.^[^ ^23^ ^]^ This physical aspect is the basis for these long lasting afterglow luminescence reactions, as precipitated reactive intermediates partake in delayed reactions due to the shifting of their physical microenvironments. In this study, we explore ways to draw out this afterglow phenomenon by means of greater production of reactive intermediates, mitigating microenvironment shifts, or a combination of the two.

Here, we expand radical electroprecipitation chemiluminescence^[^ ^10^ ^]^ to the fossilization of tris(2–2′‐bipyridyl)ruthenium(I) ([Ru(bpy)_3_]^•+^). Historically, the ECL emission has been dampened by the low lifetime and reactivity of [Ru(bpy)_3_]^•+^.^[^ ^24^, ^25^ ^]^ We achieve this by adsorbing water microdroplets, surrounded by a continuous phase of 1,2‐dichloroethane (1,2‐DCE) with 100 mM tetrabutylammonium perchlorate (TBAP), on a glassy carbon macroelectrode. The co‐reduction of tris(2–2′‐bipyridyl)ruthenium(II) ([Ru(bpy)_3_]^2+^) in the water phase and benzoyl peroxide (BPO) in the 1,2‐DCE phase create a clear precipitate on the electrode. Control experiments to evaluate this precipitate show that it is a salt of [Ru(bpy)_3_]^•+^ and the benzoate anion. The precipitation freezes the reactive [Ru(bpy)_3_]^•+^, allowing its use upon dissolution. The luminescent afterglow happened for hundreds of seconds after potential arrest, resulting in a 10^3^ enhancement in the apparent afterglow chemiluminescence lifetime. This is due to a difference in solubility of the luminophore and coreactant in the aqueous and organic phases, respectively. Additionally, we define afterglow chemiluminescence lifetime as the time from when the potential is stopped to the time light emission is not detected by the detector. These results emphasize the importance of multiphase droplet environments in the electroprecipitation of reactive intermediates. We detail how the fossilization of such intermediates can be used to extend the luminescent lifetime of classical ECL reactions, effectively separating the “E” from the “CL” in space and time.

## Results and Discussion

**Figure 1:**
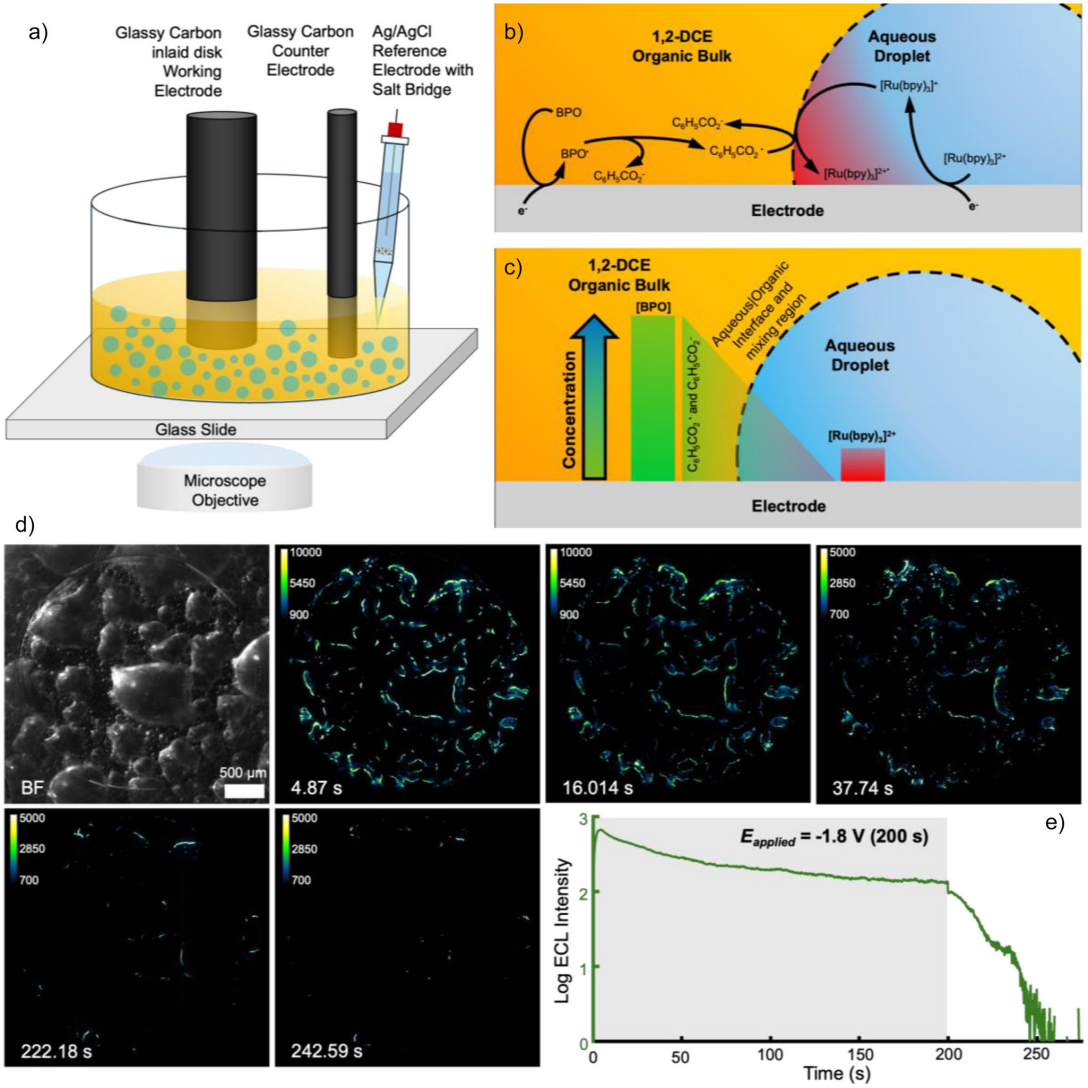
a) Experimental schematic showing the glassy carbon (GC) inlaid disk working electrode, GC rod counter electrode, and Ag/AgCl reference electrode in a glass pipette salt bridge. The opto‐electrochemical cell is composed of a glass cylinder epoxied onto a glass slide. The 4*x* microscope objective is focused onto the surface of the working electrode. b) Reaction mechanism showing the co‐reduction of benzoyl peroxide (BPO) and tris(2,2′‐bipyridyl) ruthenium(II) ([Ru(bpy)_3_]^2+^). The BPO is selectively soluble in the 1,2‐dichloroethane (1,2‐DCE) bulk solution, while the [Ru(bpy)_3_]^2+^ is primarily soluble in the aqueous droplet phase. Then homogenous reactions occur to form the excited state: [Ru(bpy)_3_]^2+*^ that emits light at the triple phase boundary. c) Pictorial schematic of different concentration profiles of species within the experimental design. Within the 1,2‐DCE droplet, BPO and a sparing amount of [Ru(bpy)_3_]^2+^ is present, whereas in the aqueous phase, [Ru(bpy)_3_]^2+^ is the primary species present. Based on this concentration gradient, the light‐emitting reaction will be primarily confined to the triple phase boundary. d) Pre‐experiment brightfield (BF), ECL, and afterglow chemiluminescence microscopy. The first frame is the brightfield before the reaction was run. The 2nd–4th frames display the ECL microscopy with the emission being confined to the electrode|organic|aqueous interface. The 5th and 6th frames show the afterglow chemiluminescence signal after the potential was stopped. Scale bar is 500 µm e) ECL intensity versus time trace. The intensity is plotted in a logarithmic scale, showing a steep decay in intensity after the potential was stopped with over 50 s of afterglow chemiluminescence time.

The experimental setup is similar to setups described previously by others,^[^ ^19^, ^22^ ^]^ wherein a glassy carbon (GC) inlaid disk macroelectrode (*d *= 3 mm) is used as the working electrode, a GC rod (*d* = 2 mm) is the counter electrode, and an Ag/AgCl electrode within a glass pipette agarose salt bridge (1 M KCl) is the reference electrode. The emulsions are composed of an aqueous droplet phase (200 µL) of 10 [Ru(bpy)_3_]^2+^ and 100 mM KCl, and a bulk continuous phase (5 mL) of 50 mM benzoyl peroxide (BPO) and 100 mM TBAP in 1,2‐dicholorethane (1,2‐DCE). The solution is sonicated to form the emulsion and decanted into the opto‐electrochemical cell on the inverted optical microscope (Figure 1a). See materials and methods for full experimental details. Once the emulsion settles, a reducing potential (‐1.8 V versus Ag/AgCl) is applied to the electrochemical cell. This causes the co‐reduction of BPO and [Ru(bpy)_3_]^2+^ to benzoate radical (C_6_H_5_CO_2_^⋅^), benzoate anion (C_6_H_5_CO_2_^−^), and [Ru(bpy)_3_]^•+^. (Figure 1b). The emission of [Ru(bpy)_3_]^2+*^ was ruled in by control experiments, as emission was only seen when both the luminophore and coreactant (Figures S1–S5) are present. Then, the radical coreactant and reduced luminophore conduct a homogeneous charge‐transfer to form the excited state: [Ru(bpy)_3_]^2+*^ to radiatively emit light. Based on this mechanism and our prior “phase‐resolving” results,^[^ ^19^ ^]^ this light emission should be confined to the triple phase boundary (aqueous|organic|electrode) based on solubility and qualitative concentration gradients combined with the diffuse mixing region of the 1,2‐DCE|water interface (Figure 1c). Furthermore, from a previous demonstration of the electroprecipitation of sulfate radical crystals,^[^ ^10^ ^]^ one should be able to precipitate out an afterglow chemiluminescence active precipitate at this triple phase boundary. The lifetime of the benzoate radical is ca. 250 ns,^[^ ^26^, ^27^ ^]^ and the lifetimes of the + 1 and + 3 oxidation state are also short (hundreds of ms, Figure S6). Therefore, based on the camera parameters of a 116 ms exposure, there should be a maximum of 2–3 frames after the potential is ended that shows emission if there is no precipitation occurring at the phase boundary. This is indeed what is seen in bulk when a control containing just the organic phase is conducted (Figure S6). By exceeding the solubility limit of a [Ru(bpy)_3_]^•^[C_6_H5O] precipitant, one should be able to extend the ECL lifetime by orders of magnitude based on the solubility of the luminophore and coreactant in the different phases. Simply, this is due to the order of magnitude difference in solubility of the luminophore in the aqueous (10 mM) and organic (1 mM) phases, which is combined with the difference in solubility of BPO in the organic and aqueous phases (>250 mM versus <<1 mM), respectively. Thus, precipitation becomes quite favorable at the triple‐phase boundary. This qualitative model (Figure 1c) is the foundation of this work by combining electrode|liquid|liquid phase boundary ECL with electroprecipitation to expand the luminescence lifetime of this reductive–oxidative organic ECL system.

The ECL microscopy (Figure 1d) supports the model with the 1,2‐DCE|water interface emitting light beyond the 200 s of applied potential for another ca. 50 s (Figure 1d,e). There is a multiple order‐of‐magnitude decrease in intensity through the 50 s, which supports a dissolution of the precipitant.

**Scheme 1:**
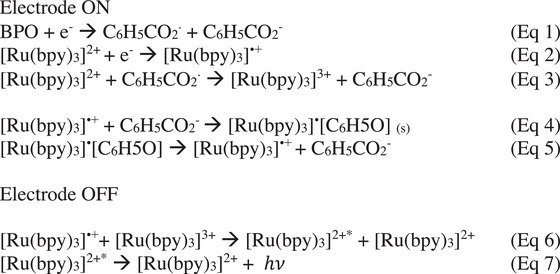
Proposed mechanism for annihilation.^[^ ^28^ ^]^

Herein, we propose a reasonable afterglow chemiluminescence mechanism of emission based on previous literature of this BPO and [Ru(bpy)_3_]^2+^ ECL system.^[^ ^28^ ^]^ To begin, BPO is reduced to its radical form, which then decomposes spontaneously to radical benzoate and benzoate anion (Eq. 1, Figure S2). Simultaneously, the reduction of the luminophore occurs to form the + 1 oxidation state (Eq. 2, Figure S4). Then, a charge‐transfer can occur between the radical coreactant and the original form of the luminophore (+2 oxidation state) to form the + 3 oxidation state (Eq. 3). This is an important step that will cause the rise of an emission known as “reductive‐oxidative” ECL (or oxidative (C_6_H_5_COO^•^)) ECL along with the comprotionative excitation, which forms the oxidized luminophore even when a cathodic potential is being applied.^[^ ^14^, ^28^ ^]^ We want to highlight that this is different than that of what was explained in Figure 1c; however, both mechanisms may occur during ECL emission depending on the emission pathway being annihilated (described here in Scheme 1) or coreactant‐mediated (Figure 1c). At the triple‐phase boundary, there is significant concentrations of both [Ru(bpy)_3_]^•+^and C_6_H_5_CO_2_^−^, thus, a precipitation may occur (Eq. 4). After the potential is stopped, the precipitant may slowly dissolve (Eq. 5) to release the + 1 oxidation state of the luminophore. The + 1 and + 3 oxidation states of the luminophore can then conduct an annihilation charge‐transfer to form the excited state that can emit a photon (Eq. 6 and Eq.7).

**Figure 2:**
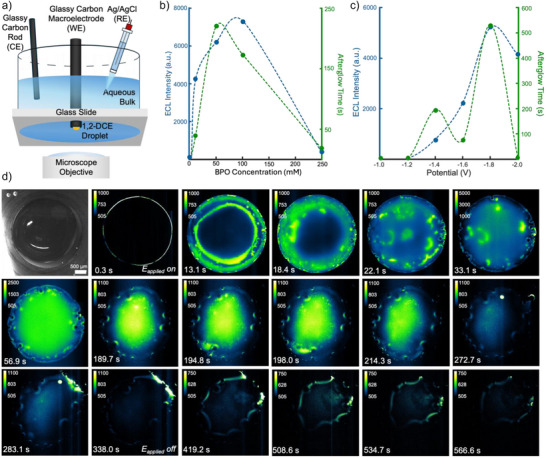
a) Experimental schematic of the hanging droplet setup. A 2 µL droplet is placed on an inlaid GC disk macroelectrode. The same reference and counter electrode stated in Figure 1 are used. b) ECL intensity (left *y*‐axis, blue) and afterglow (chemiluminescence) time (right *y*‐axis, green) based on BPO concentrations from 1 to 250 mM. The applied potential (versus Ag/AgCl) is ‐1.7 V. The maximum afterglow chemiluminescence time occurs at 50 mM, and the maximum ECL intensity occurs at 100 mM. As concentration increases, past the maximum for each, there is a decrease for both characteristics. c) ECL intensity (left *y*‐axis, blue) and afterglow chemiluminescence time (right *y*‐axis, green) based on potential (versus Ag/AgCl) from ‐1.0 to ‐2.0 V in increments of 0.2 V. The BPO concentration used is 50 mM. At both ‐1.0 and ‐1.2 V, there was no appreciable ECL or afterglow chemiluminescence. At ‐1.8 V, both the ECL intensity and afterglow chemiluminescence time were maximized. d) Brightfield and ECL microscopy of a 2 µL droplet containing 1 mM [Ru(bpy)_3_]^2+^, 50 mM BPO, 100 mM TBAP in 1,2‐DCE. The bulk phase is composed of 10 mM [Ru(bpy)_3_]^2+^ and 100 mM KCl in water. The reducing ‐1.8 V (versus Ag/AgCl) potential is applied for 300 s. The afterglow chemiluminescence time lasted for nearly 270 s. Scale bar is 500 µm.

Our investigations are rooted in the competition between electrogenerated reactive intermediate lifetime and building up a high enough concentration in a certain amount of time to exceed the solubility product equilibrium. To rule in whether C_6_H_5_COO^•^ or [Ru(bpy)_3_]^•^[C_6_H5O] was being precipitated two spike‐in experiments were performed (Figure S7). To accomplish this, similar to Figure 2, an organic droplet was placed on a glassy carbon inlaid disk macroelectrode. In the first trial, the droplet had 50 mM BPO, and the aqueous phase had 100 mM KCl (both without luminophore present). Bulk electrolyzed [Ru(bpy)_3_]^•+^ was spiked in and there was no light emission. This indicates that there is not a direct mechanism where the + 1 oxidation state of the luminophore is oxidized by the benzoate radical to form the + 2‐excited state of the luminophore. In the second experiment, the 1,2‐DCE droplet contained 50 mM BPO, 1 mM [Ru(bpy)_3_]^2+^, 100 mM TBAP, while the aqueous bulk contained 100 mM KCl. The potential (‐1.8 V versus Ag/AgCl) was applied again for 60 s. This then was allowed to go dark for 5–10 s after the potential was stopped. Then, following a spike of bulk electrolyzed [Ru(bpy)_3_]^3+^, intense light emission was observed. Thus, our precipitate likely contains [Ru(bpy)_3_]^•+^ as light emission was seen, which we postulate is caused by the annihilation mechanism, while not classically written as a radical [Ru(bpy)_3_]^•+^ is a radical on a ‐bipyridyl ligand.^[^ ^29^, ^30^ ^]^ Therefore, this precipitation is still radical precipitation, and more broadly a reactive intermediate precipitation, due to the co‐reduction of the two species. To place this work into context, others have observed long‐lasting chemiluminescence emission in ionic liquids and have postulated that the coulombic stabilization of charged intermediates proximal to the electrodes surface from ionic structures in the double‐layer is the culprit responsible for the emission time.^[^ ^31^, ^32^ ^]^

To examine a large electrode|aqueous|organic interface, a 2 µL droplet was placed on the surface of the inlaid disk GC macroelectrode. Additionally, 1 mM of [Ru(bpy)_3_]^2+^ was loaded into the droplet. All other species in the solutions and the electrodes used remained the same (Figure 2a). The two major contributors to the precipitation are the BPO concentration and the applied potential to the solution. The BPO concentration is intrinsically important since it is the radical‐forming coreactant responsible for the excitation of the luminophore, and it participates in the proposed mechanism. To examine the effects of the BPO concentration on both the ECL intensity and afterglow chemiluminescence time, different trials were conducted with the hanging droplet ranging from 1 to 250 mM BPO in the droplet phase. We chose the hanging droplet geometry due to the expanded, singular 1,2‐DCE|water interface compared to the emulsion trial shown in Figure 1d. The ECL intensity showed a maximum at ca. 100 mM BPO, and the afterglow chemiluminescence time showed a maximum at 50 mM (Figure 2b). There was a decay in ECL intensity due to the likely decarboxylation of the benzoate anion to form carbon dioxide and phenyl radicals at a higher concentration, which was reflected through analysis of the ECL signal, displaying little bubble production at lower concentrations compared to higher ones (Figure S8).^[^ ^33^, ^34^, ^35^ ^]^ The formation of additional carbon dioxide gas consequentially consumes electrode space, reducing the efficiency of the reaction. The phenyl radical (Ph^•^) lacks sufficient reducing power to efficiently generate the excited [Ru(bpy)_3_]^2^^+^* required for light emission,^[^ ^36^ ^]^ and, in fact, structurally similar aromatic radicals (e.g., phenolic compounds) have been experimentally shown to quench the [Ru(bpy)_3_]^2^^+^ ECL signal rather than enhance it.^[^ ^37^ ^]^ Furthermore, two phenyl radicals would dimerize quickly to biphenyl, which would not participate in the reaction.^[^ ^38^, ^39^ ^]^

The influence of applied potential was investigated by conducting different trials with the hanging droplet geometry, applying between −1.0 and −2.0 V (versus Ag/AgCl) in increments of −0.2 V. At −1.0 and −1.2 V (versus Ag/AgCl), there was no significant ECL emission nor afterglow chemiluminescence observed (Figure 2c). There is a local maximum for afterglow chemiluminescence time at −1.4 V (versus Ag/AgCl), while both the ECL intensity and time show a maximum at −1.8 V. For both characteristics, at −2.0 V (versus Ag/AgCl), there is a decrease, with the being eliminated. This is likely due to a competitive reaction,^[^ ^40^, ^41^ ^]^ namely hydrogen evolution reaction (HER), disturbing the interface and “knocking” the precipitate off the surface.

It is also worth noting that the lifetime of observed afterglow may be shown to be dependent on the duration of the application of potential on the system. Using the optimized potential of −1.8 V (versus Ag/AgCl) and BPO concentration of 50 mM, we then tested varying polarization times including 10, 30, 60, 120, and 180 s (Figure S9) to determine the polarization duration‐dependency of the afterglow chemiluminescence. This testing revealed that the duration of afterglow chemiluminescence is indeed dependent on polarization time, showing a positive correlation between the two variables. Given these results, we determined that our previously selected potential time of 200 seconds was suboptimal, and we promptly extended it to 300 s for future experimentation.

Given the results of the BPO concentration (Figure 2b), potential (Figure 2c), and polarization time sweeps, an optimized system of 50 mM BPO and a potential applied at ‐1.8 V (versus Ag/AgCl) for 300 s was determined. With this system, the ECL microscopy shows that initially the ECL signal was confined to the edge of the droplet (Figure 2d). Then, the spatial location of emission moves through the droplet as interfaces shift from bubble evolution and inclusion dynamics. These bubbles are likely a mixture of hydrogen from HER and CO_2_ from the decarboxylation process, discussed previously. From 56.9–214.3 s, there is a bubble that traces along the surface of the electrode. This shows that while bubbles at this potential do not necessarily move quickly, they are still dynamic at longer time scales. The afterglow chemiluminescence shows the film delaminating, or “peeling off,” the electrode surface (Supporting Information Movie 2). This is a unique phenomenon compared to what we observed previously with the persulfate radical precipitation appearing more as crystals both during growth and as they redispersed into the solution.^[^ ^10^ ^]^ We hypothesize, for future work, that this dynamic delamination is caused by the cessation of applied potential, causing mechanical perturbation. Furthermore, while we work to ensure solution quiescence, it is possible for the precipitate to be removed by some external mechanical perturbation.^[^ ^10^, ^40^ ^]^

This delamination phenomenon allows us to better understand the true lifetime of the film that is being formed because it does not fall back into solution.^[^ ^10^ ^]^ By coupling the delamination phenomenon with the optimized system based on variable sweeps (Figure 2b,c), we can estimate the magnitude increase of the afterglow chemiluminescence time versus the expected lifetime. The expected lifetime was derived from the duration of the benzoate radical, charge transfer, and the lifetimes of the luminophore in its different oxidation states based on controls (Figure S7). Benzoate radicals have been observed through time‐resolved electron spin resonance to last about 250 ns,^[^ ^26^, ^27^ ^]^ and [Ru(bpy)_3_]^2+^ in its excited state has been seen to last ca. 400–600 ns in organic solutions. Moreover, different oxidation states of [Ru(bpy)_3_]^2+^ can last for potentially hundreds of ms.^[^ ^42^ ^]^ These assumptions are validated by the control that exhibited a 0.24 s afterglow chemiluminescence time in bulk organic (Figure S6). From here, we can simply calculate the difference between the estimated bulk solution‐phase afterglow chemiluminescence lifetime (ca. 240 ms) with the droplet phase luminescence lifetime (525 s) of afterglow chemiluminescence time in the idealized system. This is then set over the bulk solution lifetime and multiplied by 100%. This estimation results in a ≈ 220000% increase in luminescence lifetime with this electroprecipitation reaction. This corresponds to a ca. 3.3 order of magnitude increase in luminescence lifetime when the ratio of the multiphase afterglow chemiluminescence time to bulk afterglow chemiluminescence time has a log_10_ applied to it.

**Figure 3:**
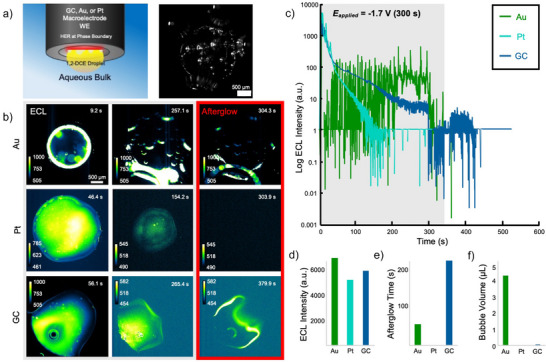
**a)** Experimental schematic of hanging droplet experiment. The working electrode is an inlaid disk macroelectrode (GC, Au, or Pt), and the other electrodes are the same as described before (not depicted). An episcopic brightfield image of the bubble evolution is shown to the right after the ECL reaction is concluded on an Au inlaid disk macroelectrode. Scale bar is 500 µm. **b)** ECL (1st and 2nd column) and afterglow chemiluminescence (3rd column) micrographs of an Au (top row), Pt (middle row), and GC (bottom row) macroelectrode. Scale bar is 500 µm. **c)** Corresponding to (B) ECL intensity versus time traces for all 3 electrodes. The *y*‐axis is on a logarithmic scale. **d)** Maximum ECL intensity during the trial for Au, Pt, and GC macroelectrode. **e)** Afterglow chemiluminescence time during the trial for Au, Pt, and GC macroelectrodes. **f)** Evolved bubble volume during the trial for Au, Pt, and GC macroelectrode. Figure S11 shows the overlay images, assumptions, and method for calculating the volumes based on our previous through‐space work.^[^ ^18^ ^]^

Competitive reactions, such as HER, are tunable with careful selection of electrode material. To investigate this, the same hanging droplet setup as before was used, this time changing only the material of the inlaid‐disk electrode (Figure 3a). To exemplify this phenomenon of increased HER, a brightfield image of the gold macroelectrode after the ECL reaction was performed was captured, and it exhibited many bubbles on the surface of the electrode (Figure 3a). The electrode materials tested showed notable differences in precipitation patterns and HER favorability, both of which were clearly visible in microscopy, both during and after potential was applied (Figure 3b **, Au**). Gold showed slight precipitation and afterglow chemiluminescence that lasted about 50 s after potential was stopped, but overactive bubble dynamics prevented any significant precipitation from occurring. Gold, being largely chemically inert, is not anticipated to remove atomic hydrogen from the system, which is likely why a large amount of gas was produced in this trial.

Platinum yielded surprisingly low emission, so much so that the emission did not last the duration of the application of potential (Figure 3b **, Pt**). Further investigation of this phenomenon included testing for reproducibility and utilization of a 40*x* objective (Figure S10). The brightfield showed neither significant bubble growth nor electroprecipitation; thus, a reasonable hypothesis to explain this phenomenon is that the surface of the electrode is being modified and blocked by platinum hydride formation.^[^ ^43^, ^44^, ^45^, ^46^ ^]^ Given the surprisingly low volume of gas produced through HER on platinum as compared to gold (Figure 3f), this is a reasonable conclusion. Platinum, although still mostly chemically inert, has been shown to form these hydrides, which consume the electrode surface rather than share it, and, hence, we hypothesize that these formed hydrides account for the reduced ECL time and negligible afterglow time.

GC saw the clearest precipitation and longest‐lasting afterglow chemiluminescence (Figure 3b, **GC**, 3c, 3e). It is for this reason that we selected GC as our electrode for the previously discussed sweeps of BPO concentration and applied potential. All three electrodes displayed similar max mean intensities (Figure 3d).

**Figure 4:**
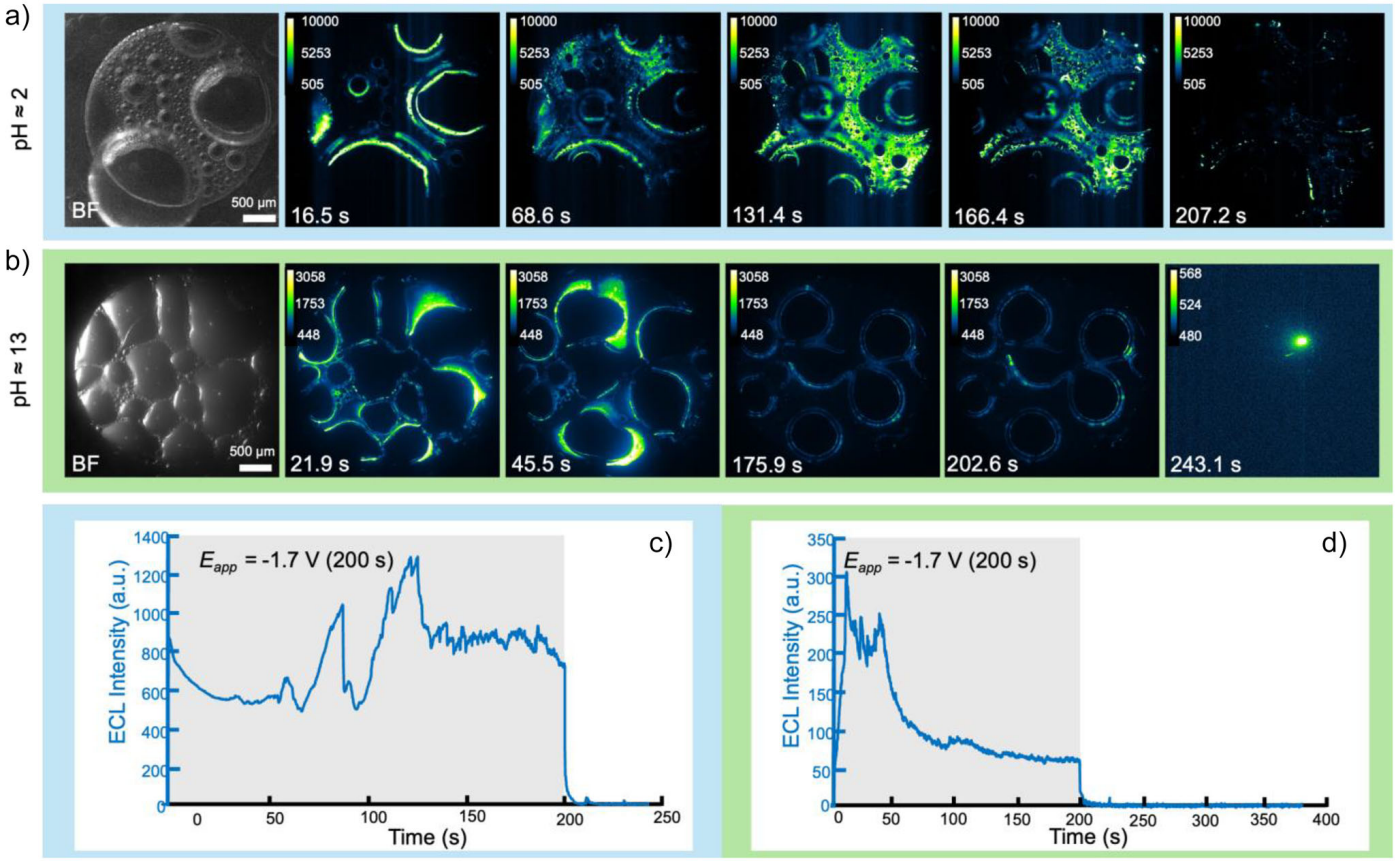
**a)** Pre‐experimental brightfield, ECL, and afterglow chemiluminescence microscopy of the acidic emulsion settled on an inlaid‐disk GC electrode. The droplet phase consists of 10 mM [Ru(bpy)_3_]^2+^ and 100 mM KCl in water, which had been spiked with several drops of 1 M HCl to lower the pH to approximately 2. The bulk phase contains 50 mM BPO and 100 mM TBAP in 1,2‐DCE. Phases were emulsified in a 1:25 ratio (200 µL and 5 mL, respectively). Scale bar is 500 µm. **b)** ECL intensity versus time trace corresponding to the trial in (a). **c)** Pre‐experimental brightfield, ECL, and afterglow chemiluminescence micrographs of the basic emulsion, settled on an inlaid‐disk GC electrode. The droplet phase contains 10 mM [Ru(bpy)_3_]^2+^ and 100 mM KCl in water, this time spiked with several drops of 1 M NaOH to increase the pH to approximately 13. The bulk phase is composed of 50 mM BPO and 100 mM TBAP in 1,2‐DCE. Phases were emulsified in a 1:25 ratio (200 µL and 5 mL, respectively). Scale bar is 500 µm. **d)** ECL intensity versus time plot corresponding to the trial in (c). The electrochemistry (amperometric i–t curves holding ‐1.8 V (versus Ag/AgCl)) for these two trials and cyclic voltammetry of 100 mM KCl in pH 2 and pH 13 solutions is presented in Figure S12.

Further tunability of HER can be achieved by adjusting the pH of the aqueous droplet phase in an emulsion. Lowering the pH of the aqueous phase to a pH of ca. 2 (Figure 4a) led to a highly increased volume of gas produced not only at the triple phase boundaries but also at the electrode|aqueous interface as well. The volume of gas produced in this trial reached a point at which the afterglow chemiluminescence time was inhibited, shown by the trial's corresponding intensity‐time plot's steep decline after 200 s (Figure 4c). Similarly, increasing the pH of the aqueous phase reduces the amount of gas produced, almost completely eliminating it (Figure 4b). This trial additionally showed extended afterglow chemiluminescence, with slight, occasional flashes for 1–2 min after the potential ended (Figure 4d). By tuning the pH, we either promote or hinder HER in the aqueous phase. As a general comment, at acidic pHs, due to the presence of many H^+^ ions, HER is favored. This results in bubble nucleation occurring rapidly in the acidic, aqueous droplets from the high local flux of the protons (Figure S12). Whereas at basic pHs, HER is disfavored, comparatively due to water reduction needing to occur, which is sluggish, especially when considering H_2_ bubble generation. So, while both, based on the pH, will produce H_2_, bubbles will nucleate and grow in the acidic droplets. Additionally, we would like to add to the discussion regarding the decarboxylation process, which forms CO_2_. If one increases the concentrations of protons (acidic aqueous solution) carbonic acid will be favored to form, whereas in basic regimes, the opposite is true. Thus, there is a trade‐off between the two‐gas evolution reactions occurring.

Given the proposed mechanism for the reaction in place, optimizations to reduce HER activity are crucial for researching other competing reactions in this avenue of inquiry. Inhibiting HER by selecting a combination of variables discussed previously allows for the investigation of competitive reactions like decarboxylation, which would otherwise be overshadowed by the volume of hydrogen gas produced.

## Conclusion

Radicals and reactive intermediates are a hallmark of pure chemistry. There are currently very few methods to isolate such reactive species and use them for interesting chemical reactions. Our previous work has demonstrated a method to electroprecipitate highly reactive radicals for their use in ECL. Here, we show that ECL apparent lifetimes can be enhanced by three orders of magnitude during the co‐reduction of benzoyl peroxide and [Ru(bpy)_3_]^2+^. One requirement is a multiphase environment, where BPO is in the continuous oil phase (1,2‐DCE) and [Ru(bpy)_3_]^2+^ is confined in aqueous microdroplets adsorbed to the electrode. Co‐reduction of these molecules creates [Ru(bpy)_3_]^•+^, a radical and reactive intermediate, and the benzoate anion. Because the potential application produces the phenolate radical anion, a concentration profile of [Ru(bpy)_3_]^3+^ exists. When the potential is turned off, the [Ru(bpy)_3_]^•^[C_6_H5O] salt redissolves into the DCE phase, where [Ru(bpy)_3_]^•+^ meets a [Ru(bpy)_3_]^3+^. The enthalpy of annihilation is such that a photon can be produced from the annihilation reaction, effectively creating a long‐lived afterglow effect.

In our previous work, we demonstrated that reactive radical species could be fossilized.^[^ ^10^ ^]^ The central chemical tenet that allows for this is as follows: If a radical is created that has a certain solubility product equilibrium with a counter‐ion, the radical can precipitate if the solubility product equilibrium is exceeded before the radical lifetime. This is quite difficult to do homogeneously because such concentrations are difficult to achieve. In the current manuscript, we demonstrate that the technique is much more generalizable: Not only can we electroprecipitate radicals, we can electroprecipitate reactive intermediates in general. It is also conceivable that uncharged reactive intermediates and be co‐precipitated in a salting‐out process. While we explore multiphase systems in this work, it is worth noting that the technique can be extended across multiphase environments central to energy storage and biology.^[^ ^22^ ^]^

Our results hold significance because they reinforce the ability to electroprecipitate highly reactive species that can be used in chemiluminescence reactions. Various organic posolytes have been observed to be suboptimal as electrolytes because high oxidation potentials generally degrade, discharge, and create unreliable conditions for use in redox flow batteries.^[^ ^47^ ^]^ By exploring the reductive regime, we can observe increased reliability for longer‐lasting reactive species, which shows promise in the field of alternative energy storage.^[^ ^48^, ^49^ ^]^ Ultimately, our results demonstrate that electrochemiluminescence can be separated in space and time, electroprecipitating reactive intermediates that can be used, in principle, whenever and wherever. As the global demand for biosensors and energy storage and conversion devices continues to rise, reactive intermediate chemistry is a cornerstone when it comes to understanding important interfacial processes.

## Author Contributions

Daniel M. Carrel, Brady R. Layman, Megan L. Hill, and Jeffrey E. Dick designed experiments. Daniel M. Carrel, Brady R. Layman, and Megan L. Hill performed all experiments. Daniel M. Carrel, Brady R. Layman, and Megan L. Hill analyzed all data. The manuscript was written from the contributions of all authors. Jeffrey E. Dick supervised all aspects of the research and was responsible for funding acquisition.

## Conflict of Interests

The authors declare that they have filed a provisional patent on this technology.

## Supporting information



Supporting Information

Supporting Information

Supporting Information

Supporting Information

Supporting Information

Supporting Information

Supporting Information

Supporting Information

## Data Availability

The data that support the findings of this study are available from the corresponding author upon reasonable request.
