# Supplementary material for: Radical Electroprecipitation from a Water|Oil|Electrode Interface Prolongs “Electro”Chemiluminescence of the Tris(2,2′‐bipyridyl)Ruthenium(II) and Benzoyl Peroxide System by 103
Source: Angew Chem Int Ed Engl. 2026 Jan 7;65(7):e21454. doi: 10.1002/anie.202521454 (PMC12887639; doi:10.1002/anie.202521454)
Supplement: Supplementary file 1 — Supporting Information [file ANIE-65-e21454-s007.docx]

*Supporting Information for*

**Radical Electroprecipitation from a Water|Oil|Electrode Interface Prolongs “Electro”chemiluminescence of the Tris(2,2’-bipyridyl)ruthenium(II) and Benzoyl Peroxide System by 10^3^**

Daniel M. Carrel^a §^, Brady R. Layman^a§^, Megan L. Hill^a^, Jeffrey E. Dick^a,b^*

^a^Department of Chemistry, Purdue University, West Lafayette, IN, 47907

*To whom correspondence should be addressed: [jdick@purdue.edu](mailto:jdick@purdue.edu)

^§^Indicates Equal Contribution

| **Title:** | **Page(s)** |
| --- | --- |
| **Materials and Methods** | S2-3 |
| **Figure S1.** 100 mM KCl in water: Cyclic voltammetry and ECL micrograph | S4 |
| **Figure S2.** Bulk measurement of 50 mM BPO and 100 mM TBAP in 1,2-DCE: Cyclic voltammetry and ECL micrograph | S5 |
| **Figure S3.** 100 mM TBAP in 1,2-DCE: Cyclic voltammetry and ECL micrograph | S6 |
| **Figure S4.** Bulk measurement of 100 mM TBAP in 1,2-DCE, saturated with [Ru(bpy)_3_]^2+^: Cyclic voltammetry and ECL micrograph | S7 |
| **Figure S5.** Bulk measurement of 100 mM TBAP and 50 mM BPO in 1,2-DCE, saturated with [Ru(bpy)_3_]^2+^: Cyclic voltammetry and ECL micrograph | S8 |
| **Figure S6.** ECL and afterglow chemiluminescence intensity and correlated amperometric i-t hold (right, green) for bulk solution containing 1 mM [Ru(bpy)_3_]^2+^, 50 mM BPO, 100 mM TBAP in 1,2-DCE with a -1.8 V (vs. Ag/AgCl) potential held for 300 s. | S9 |
| **Figure S7.** Spike-in experiments for mechanism validation. | S10 |
| **Figure S8.** Bubble formation dependency on BPO concentration. | S11 |
| **Figure S9.** Afterglow Chemiluminescence Time as a function of polarization time. | S12 |
| **Figure S10.** Increased magnification on a platinum macroelectrode using a 40x objective. | S13 |
| **Figure S11.** Overlaid micrographs and example calculation of an individual bubble’s volume. | S14-15 |
| **Fisure S12.** Amperometric i-t curve and bulk Cyclic Voltammogram corresponding to Figure 4 with acidic and basic bulk water. | S16 |
| **SI Movie Descriptions** | S17 |

**Materials and Methods:**

***Materials:***

Tris(2,2’-bipyridyl)ruthenium(II) chloride hexahydrate ([Ru(bpy)3][Cl2]·6H2O, 98%), potassium chloride (KCl, certified ACS), tetrabutylammonium perchlorate (TBAP, > 99%), sodium hydroxide (NaOH, certified ACS, 97%), hydrochloric acid (HCl, certified ACS Plus, 12.1 M), and 1,2-dichloroethane (HPLC grade, > 99%) were all supplied by ThermoFisher Scientific. Luperox® A98 and benzoyl peroxide (reagent grade, ≥ 98%) were purchased from Millipore Sigma.

All chemicals were used *as-received* with no further purification steps. Before use in experiments, the hydrochloric acid was diluted to approximately 1 M. The tris(2,2’-bipyridyl)ruthenium(II) chloride hexahydrate was stored in a relatively dark place inside a nitrogen-flushed desiccator. All aqueous solutions were prepared with ultrapure water (Millipore Milli-Q, 18.2 MΩ cm). The benzoyl peroxide was stored in a refrigerator at 4ºC.

***Opto-electrochemical Cell Fabrication:***

During experimentation, solutions and emulsions were placed into glass opto-electrochemical cells, as depicted in the main text schematics. These glass opto-electrochemical cells were constructed by using Gorilla brand Two-Part Epoxy (gorillatough.com, USA) to attach a glass cylinder (Scientific Glass Blowing Lab, Purdue University Department of Chemistry) with internal diameter and height of approximately 19 mm and 20 mm, respectively, to a glass coverslip (#1 coverslip, VWR, Germany, 24 mm x 40 mm x 130-160 um). The cells were left to cure overnight before use in experiments.

***Electrochemistry:***

Any electrochemistry done was conducted with a CHI model 6284F potentiostat (CH Instruments, Austin, Texas). For all experiments, an Ag/AgCl (1M KCl) reference electrode (CH Instruments, Austin, Texas) and a salt bridge constructed using a glass Pasteur pipette and agarose with 1 M KCl was used. Additionally, all electrochemical experiments employed the use of a glassy carbon rod (d = 2 mm, L = 10 cm, CH Instruments, Austin, Texas) as the counter electrode. A variety of inlaid disk macroelectrodes, including gold (d = 2 mm), platinum (d = 2 mm), and glassy carbon (d = 3 mm) (CH Instruments, Austin, Texas), were used as working electrodes in all experiments, and the material used in each trial, if not glassy carbon, is communicated in the associated text.

Amperometry was performed on the solution within the opto-electrochemical cell in all experiments, and the parameters used were as follows: Initial E = varied between -1.0 and -2.0, and clearly labelled in each use case, sample interval (V) = 0.001, quiet time = 0 seconds, and sensitivity (A/V) = 0.01.

***Microscopy***

To collect microscopy data during experiments, a Nikon Eclipse Ti2 inverted optical microscope (Nikon, Japan) was used. Bright-field images were collected using an external 6-Watt snake light as the light source (AmScope, Irvine, California). Every experiment discussed in the main text used the 4x objective with a numerical aperture of 0.20 and a working distance of 20 mm.

The EMCCD iXon 888 camera (Andor Technology Ltd., Belfast, UK) was used in all electrochemiluminescence microscopy data acquisition. Different trials used different acquisition settings on the camera, and they are as follows:

Movie 1 (Figure 1D): 10 ms exposure time, 200 EM gain multiplier. Movie 2 (Figure 2D): 10 ms exposure time, 50 EM gain multiplier. Movie 3 (Figure 3B, row labelled “Au”): 116 ms exposure time, 0 EM gain multiplier. Movie 4 (Figure 3B, row labelled "Pt”): 116 ms exposure time, 0 EM gain multiplier. Movie 5 (Figure 3B, row labelled “GC”): 116 ms exposure time, 0 EM gain multiplier. Movie 6 (Figure 4A): 10 ms exposure time, 200 EM gain multiplier. Movie 7 (Figure 4B): 116 ms exposure time, 0 EM gain multiplier.

All trials used gain setting 2, binning disabled, and the camera had a chip temperature of about -81.9ºC.

***Specific Experimental Details:***

Emulsion experiments (Figure 1 and 4): In each of the experiments requiring study of an emulsion, a 1:25 ratio (200 µL/5 mL) of droplet/bulk phase mixture was emulsified using a model Q500 horn sonicator (Qsonica, Newtown, Connecticut) with the following parameters: 40% amplitude, 30 second total run time, 5 second pulses both on and off. The sonicator was run twice before transferring the emulsion into an opto-electrochemical cell placed in the inverted optical microscope. The emulsion was left to sit in the cell and settle for at least 30 minutes to allow for clear visibility of any droplets that settled on the macroelectrode surface. The camera was focused on the surface of the inlaid disk electrode, which is where the triple phase boundary (aqueous|organic|electrode) is located.

Hanging droplet experiments (Figure 2 and 3): In each of the experiments conducted with a single droplet, an opto-electrical cell was filled with 2 mL of aqueous solution, and a *ca.* 2 µL droplet of the organic phase was placed on the bottom of the cell, using either a 2 µL micropipette or a Microliter Syringe (Hamilton Instruments, Cinnaminson, New Jersey). After the cell was placed onto the stage of the inverted optical microscope and all the electrodes were put in place, the macroelectrode was slowly lowered onto the droplet until they contacted each other. After this, the macroelectrode was slowly raised such that the droplet adhered to the electrode rather than the bottom of the electrochemical cell. The camera was then focused on the surface of the electrode, which is where the triple phase boundary (aqueous|organic|electrode) was located.


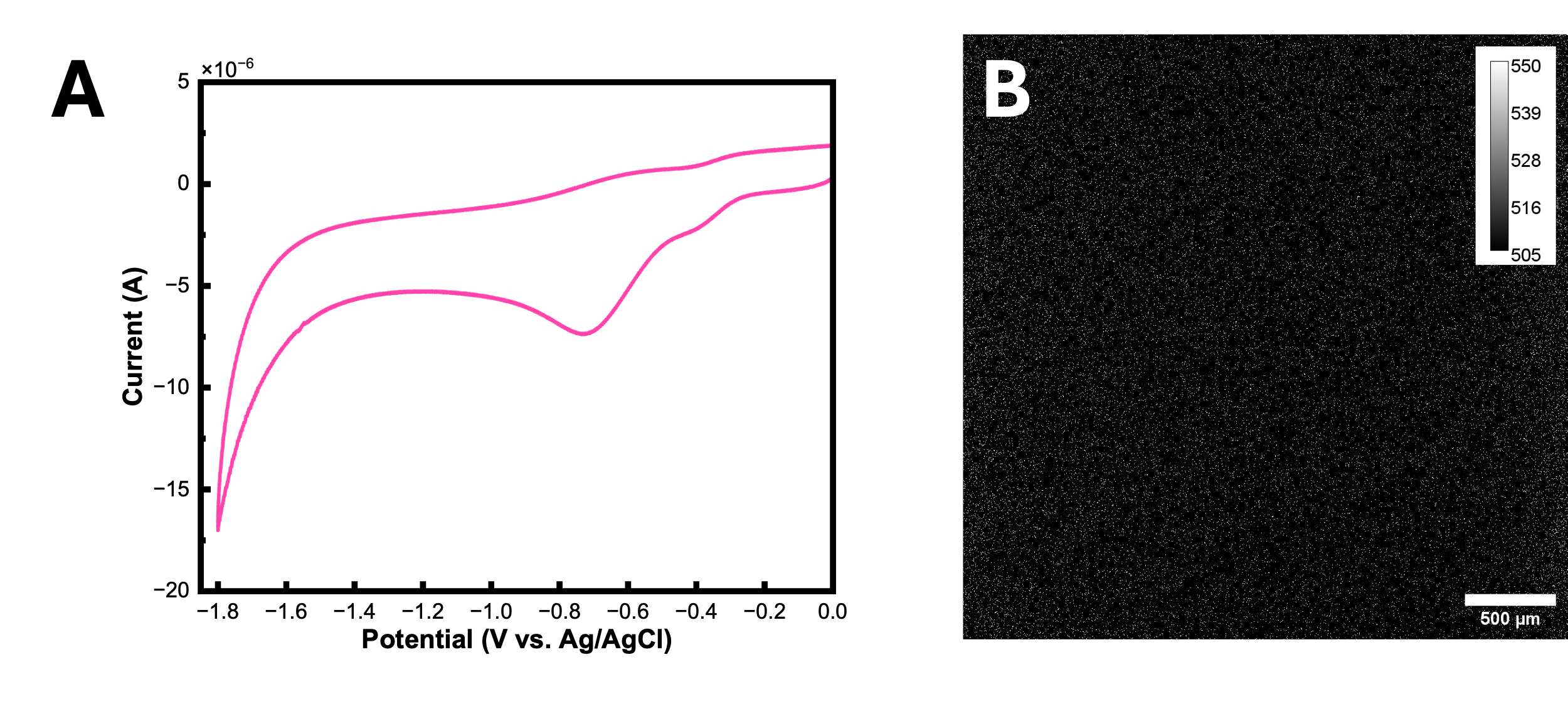


**Figure S1. 100 mM KCl in water. (A)** Cyclic voltammogram (0.0 V to -1.8 V vs. Ag/AgCl) response of the system. **(B)** ECL micrograph showing no signal.


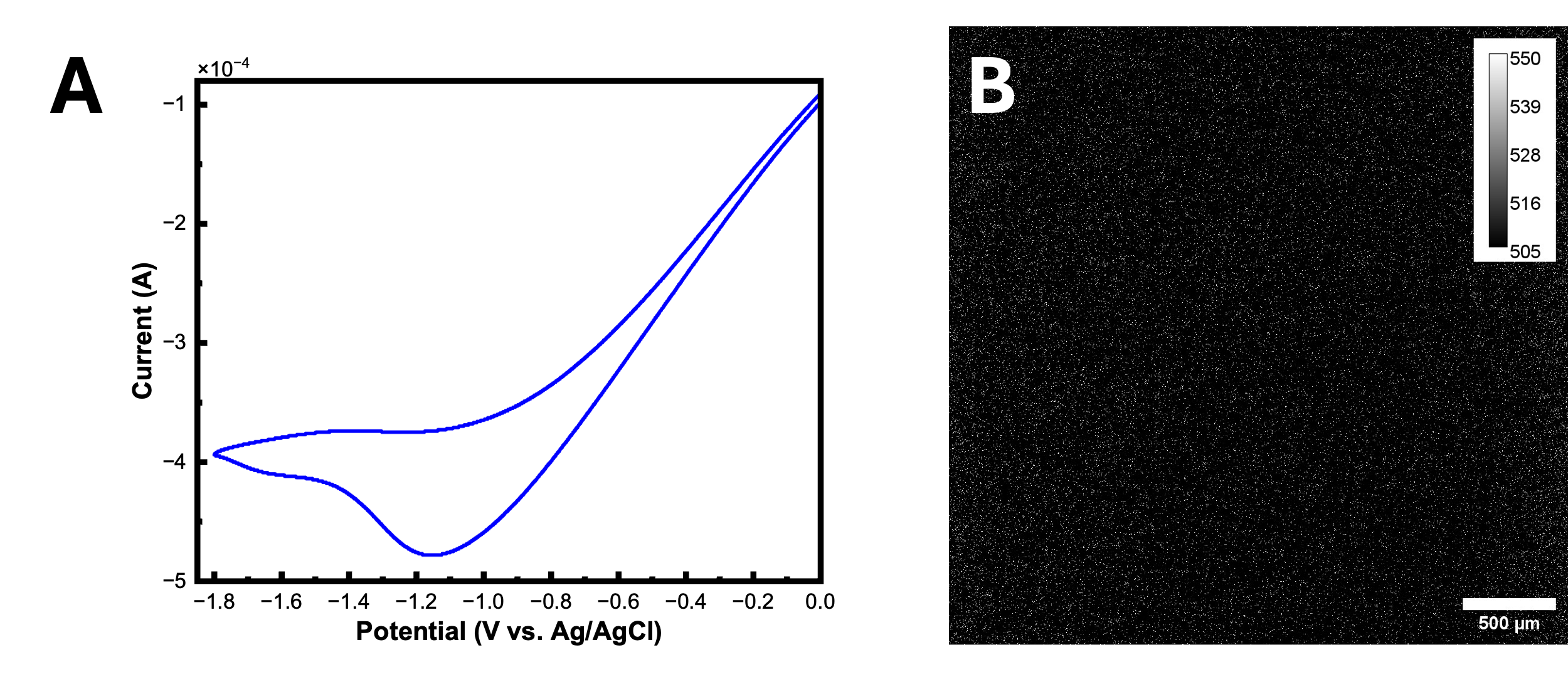


**Figure S2. Bulk measurement of 50 mM benzoyl peroxide and 100 mM tetrabutylammonium perchlorate in 1,2-dichloroethane. (A)** Cyclic voltammogram (0.0 V to -1.8 V vs. Ag/AgCl) response of the system. **(B)** ECL micrograph showing no signal.


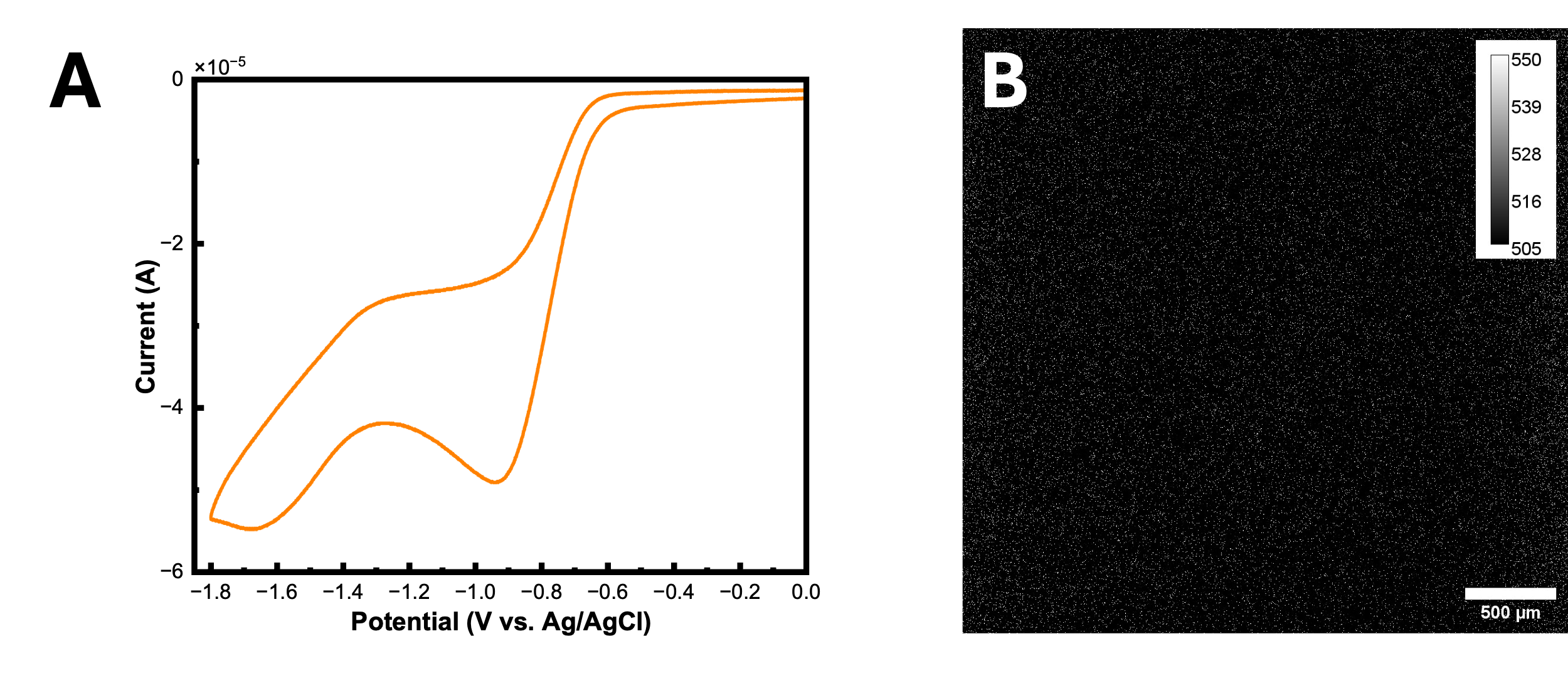


**Figure S3. Bulk measurement of 100 mM tetrabutylammonium in 1,2-dichloroethane. (A)** Cyclic voltammogram (0.0 V to -1.8 V vs. Ag/AgCl) response of the system. **(B)** ECL micrograph showing no signal.


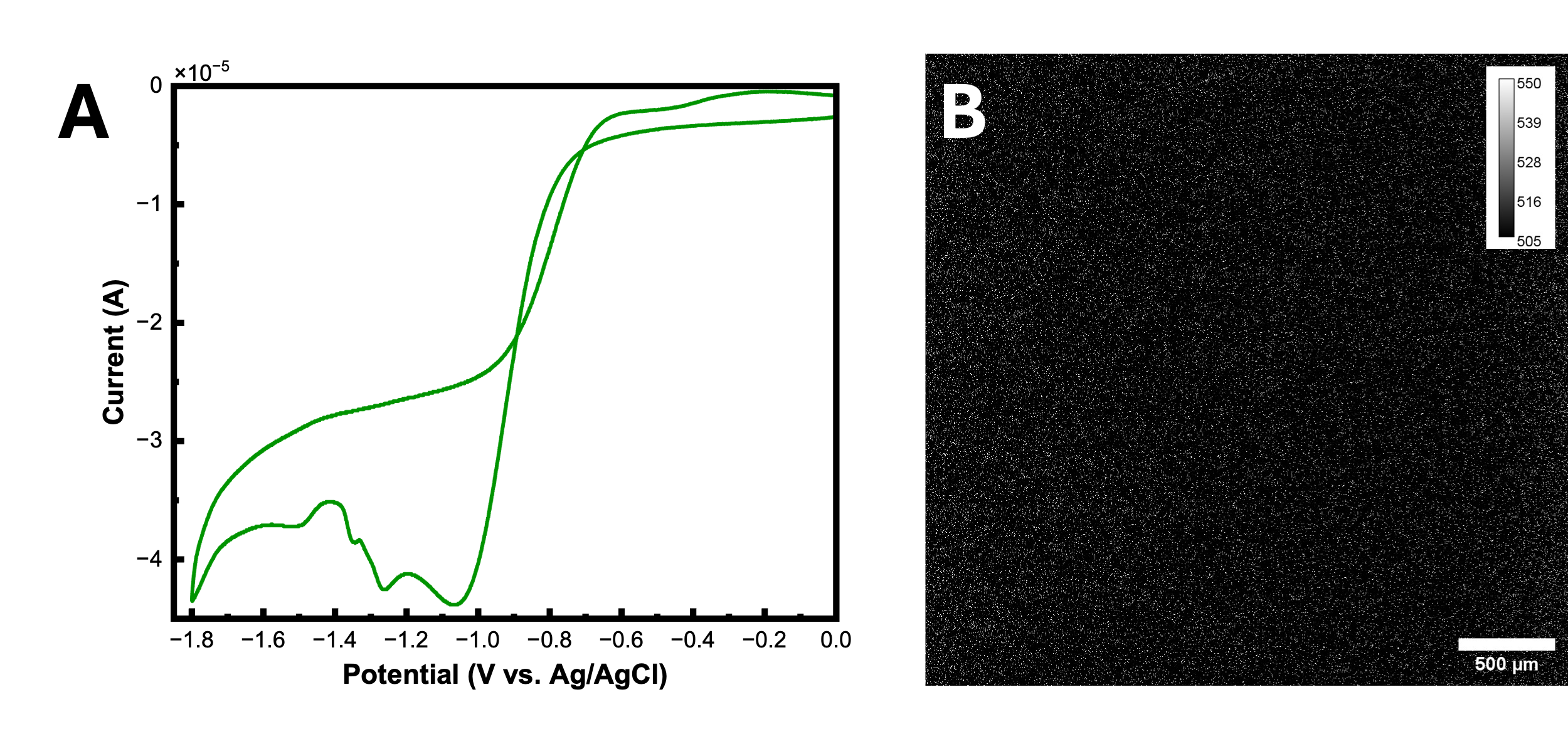


**Figure S4. Bulk measurement of 100 mM tetrabutylammonium perchlorate in 1,2-dichloroethane, saturated (*ca.* 1 mM) with tris(2,2’-bipyridyl)ruthenium(II) chloride hexahydrate. (A)** Cyclic voltammogram (0.0 V to -1.8 V vs. Ag/AgCl) response of the system. **(B)** ECL micrograph showing no signal.

**
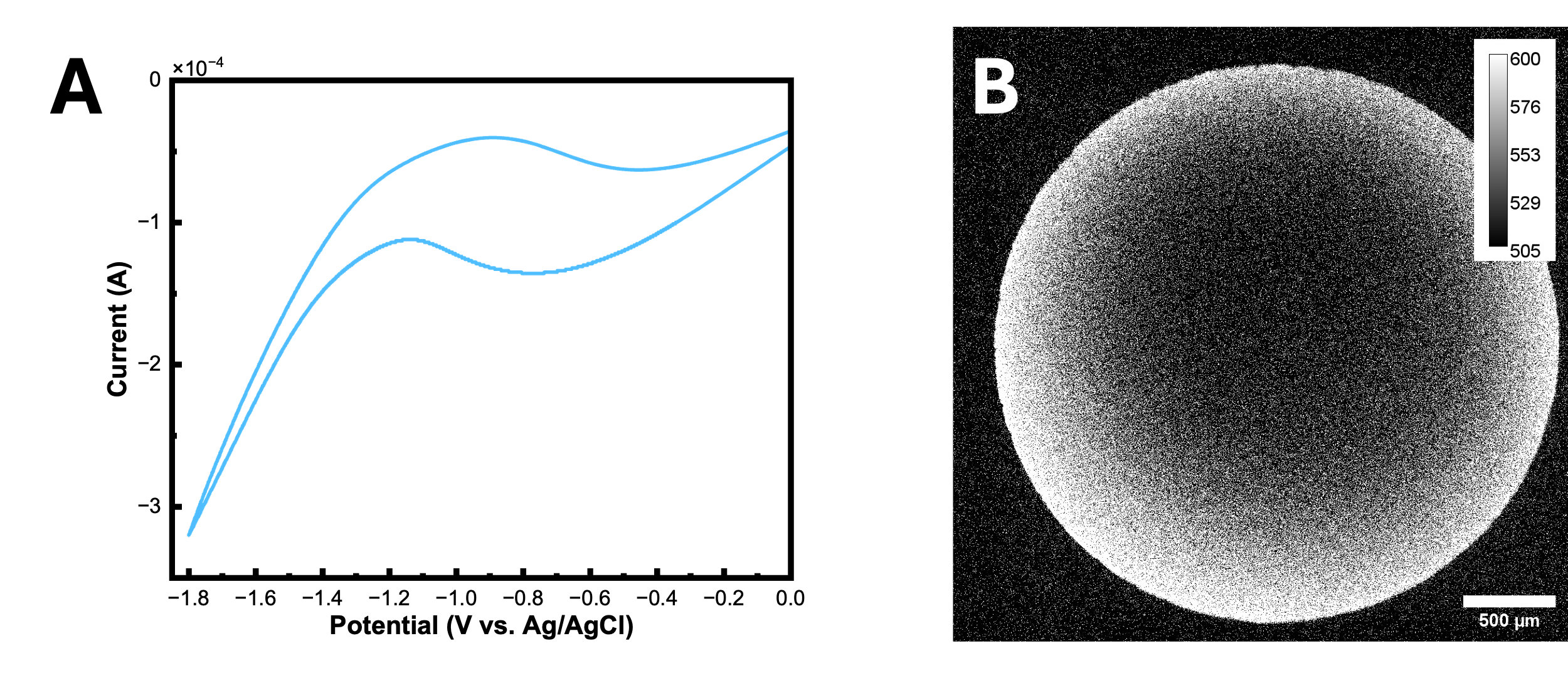
**

**Figure S5. Bulk measurement of 50 mM benzoyl peroxide and 100 mM tetrabutylammonium perchlorate in 1,2-dichloroethane, saturated (*ca.* 1 mM) with tris(2,2’-bipyridyl)ruthenium(II) chloride hexahydrate. (A)** Cyclic voltammogram (0.0 V to -1.8 V vs. Ag/AgCl) response of the system. **(B)** ECL micrograph showing positive signal.

**Figure S6. (A)** ECL intensity (left, blue) and correlated amperometric i-t hold (right, green) for a solution containing 1 mM [Ru(bpy)_3_]^2+^, 50 mM BPO, 100 mM TBAP in 1,2-DCE with a -1.8 V (vs. Ag/AgCl) potential held for 300 s. 0 represents the cameras limit of detection calculated from the mean background noise added to 3 standard deviations of noise. **(B)** Afterglow Chemiluminescence Intensity after the potential was stopped from (A). The signal is not detectable after 2 images once the potential is stopped, corresponding to a 0.224 s afterglow chemiluminescence time inherent to the system (likely due to the +1 and +3 oxidation states being present). Thus, the afterglow chemiluminescence time of bulk is 0.24 s.
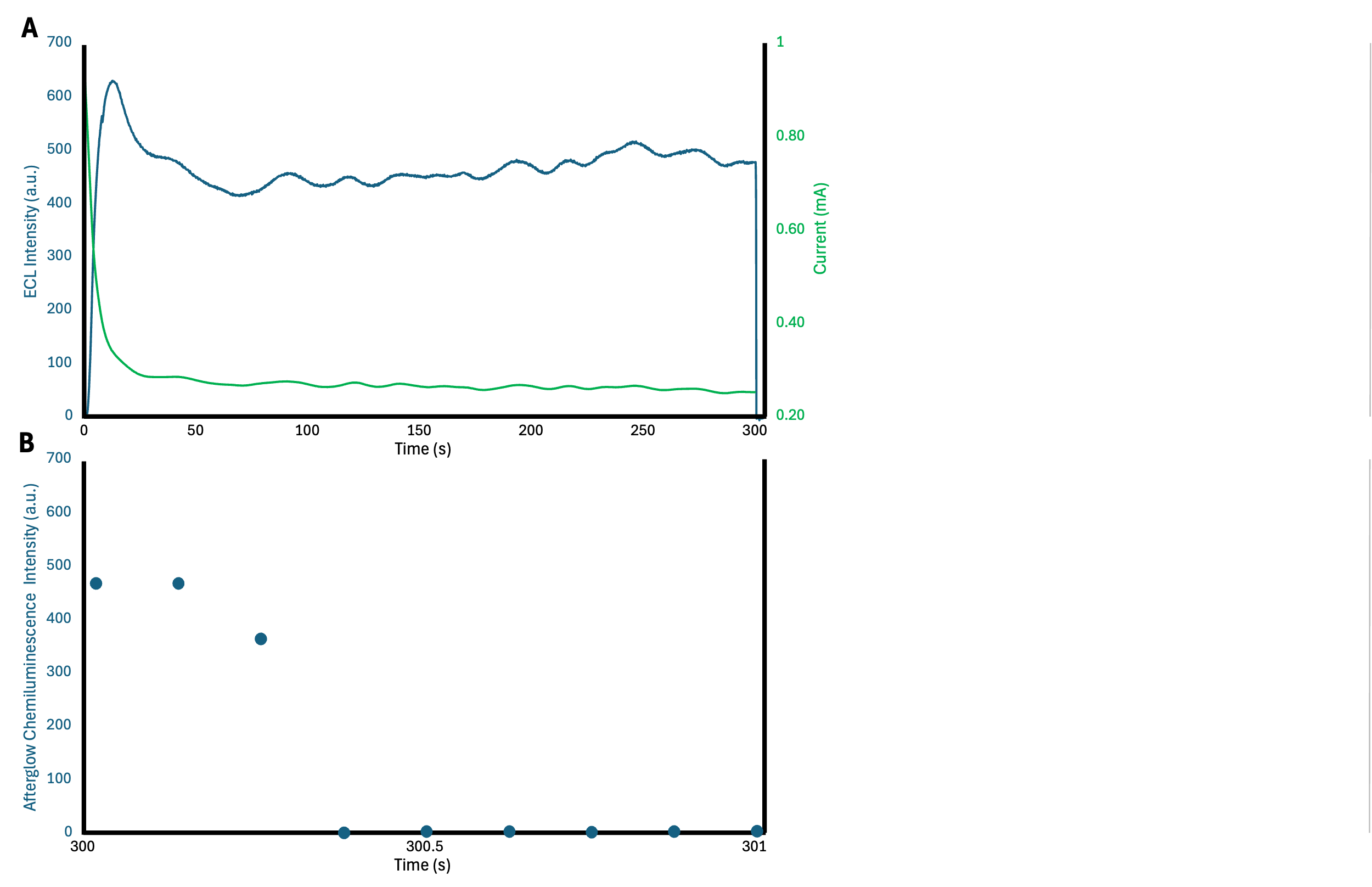


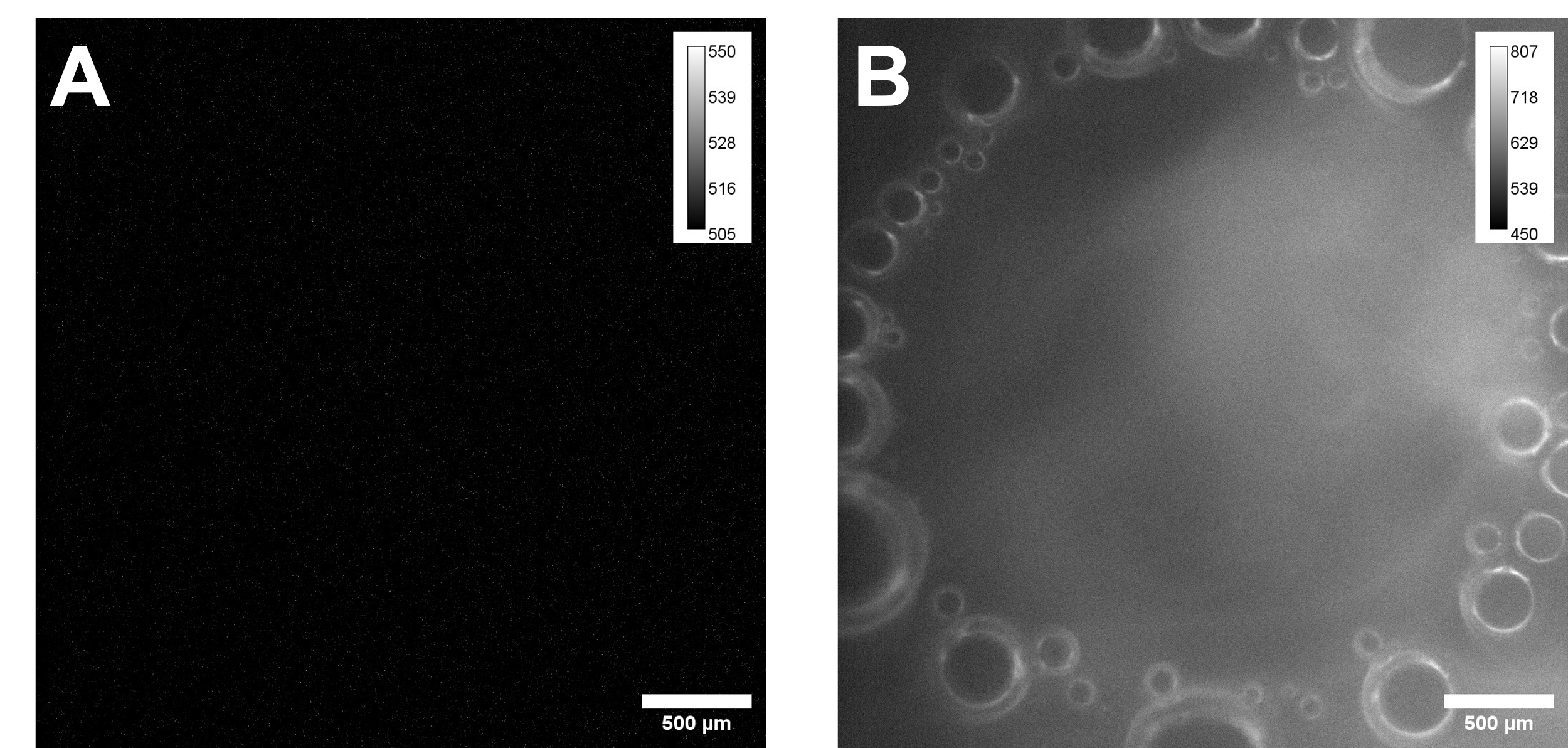


**Figure S7. Spike-in experiments for mechanism validation. (A)** Approximately 500 µL of 10 mM tris(2,2’-bipyridyl)ruthenium^1+^ and 100 mM KCl in water dispensed into the opto-electrochemical containing 100 mM KCl in water bulk phase and 1 mM tris(2,2’-bipyridyl)ruthenium(II) chloride hexahydrate, 50 mM BPO, and 100 mM TBAP in 1,2-dichloroethane droplet phase, approximately 5 seconds after potential was stopped. No afterglow chemiluminescence was observed. **(B)** Approximately 500 µL of 10 mM tris(2,2’-bipyridyl)ruthenium^3+^ and 100 mM KCl in water dispensed into the opto-electrochemical containing 100 mM KCl in water bulk phase and 1 mM tris(2,2’-bipyridyl)ruthenium(II) chloride hexahydrate, 50 mM BPO, and 100 mM TBAP in 1,2-dichloroethane droplet phase, approximately 5 seconds after potential was stopped. Afterglow chemiluminescence was observed here, validating the proposed reaction mechanism.


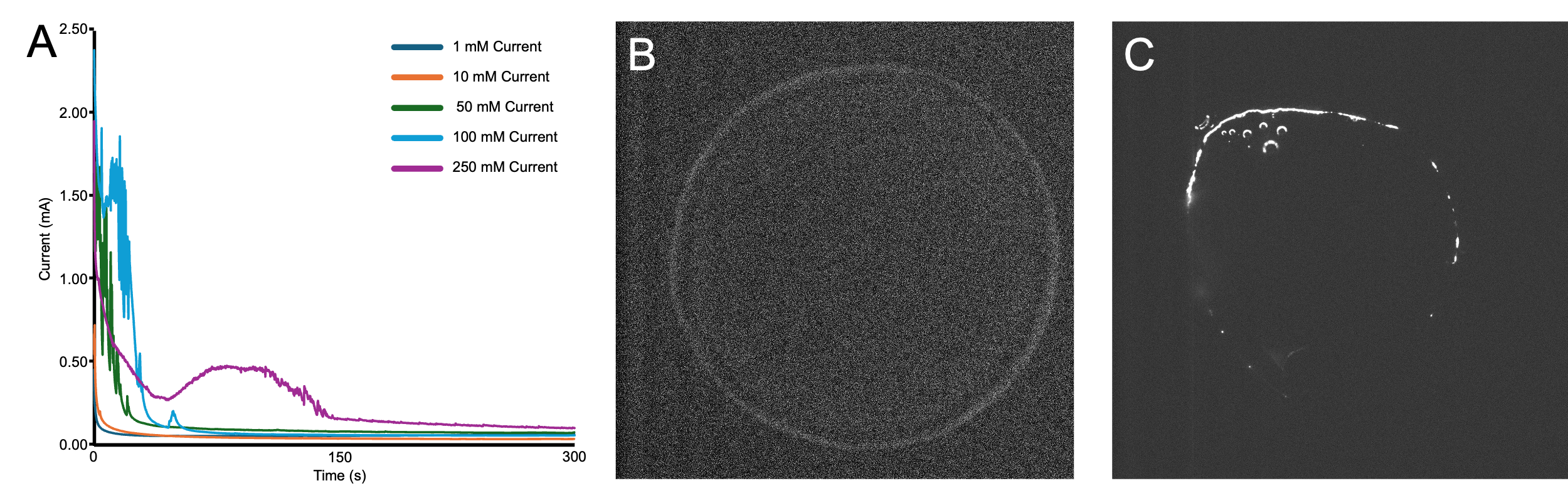


**Figure S8. Bubble formation dependency on BPO concentration. (A)** Amperometric i-t curves corresponding to each of the BPO concentrations used. Selected concentrations include 1, 10, 50, 100, and 250 mM. A potential of -1.8 V (vs. Ag/AgCl) was held. **(B)** ECL micrograph selected from the 1 mM trial, showing no bubble nucleation. **(C)** ECL micrograph selected from the 250 mM trial, showing bubble nucleation.

**
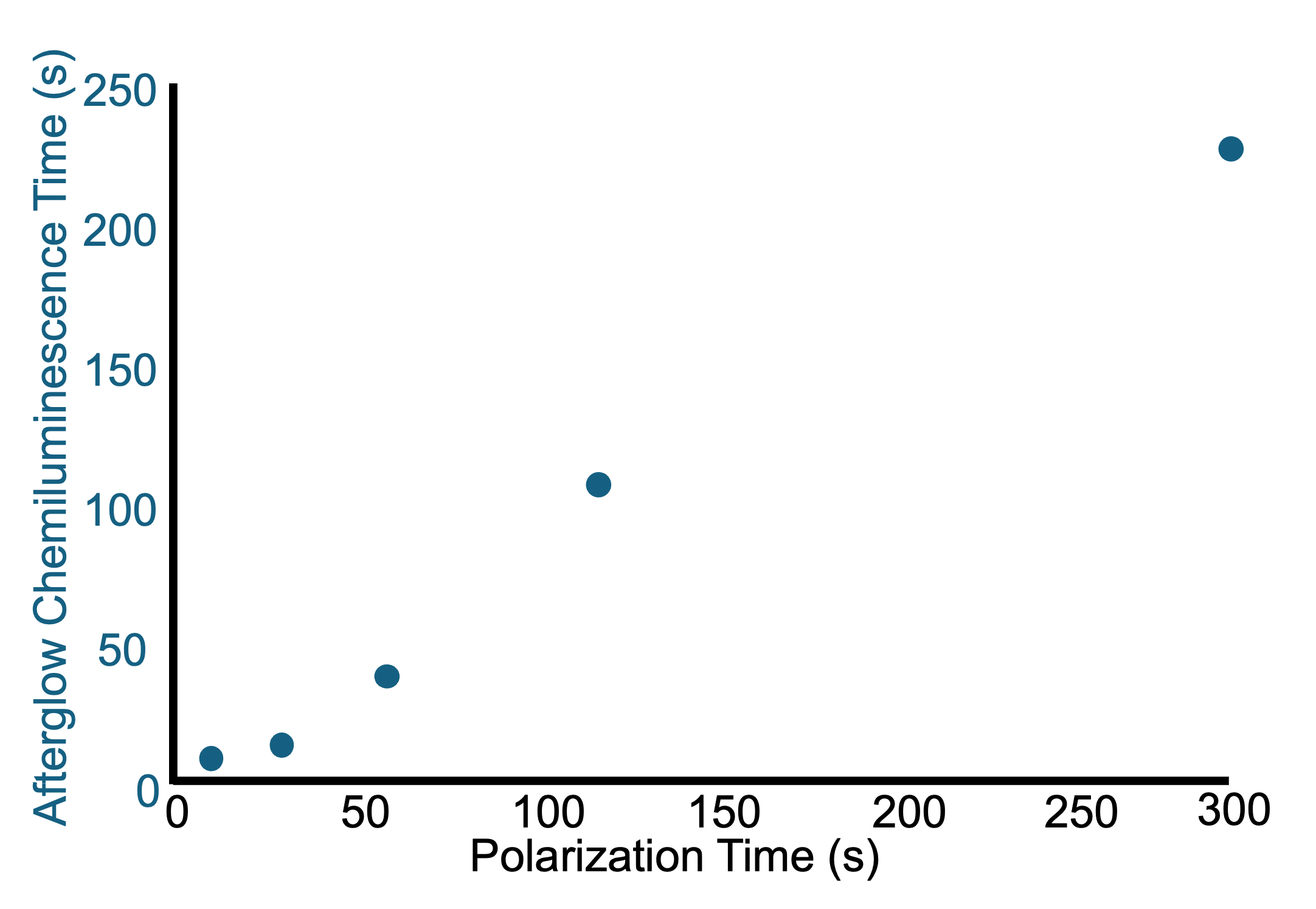
**

**Figure S9. Afterglow Chemiluminescence Time as a function of polarization time.** 2 uL of 1,2-DCE containing 1 mM [Ru(bpy)_3_]^2+^, 50 mM BPO, 100 mM TBAP, was adsorbed onto an electrode in aqueous bulk containing 10 mM [Ru(bpy)_3_]^2+^, and 100 mM KCl. A potential of - 1.8 V (vs. Ag/AgCl) was applied to the solution.

**
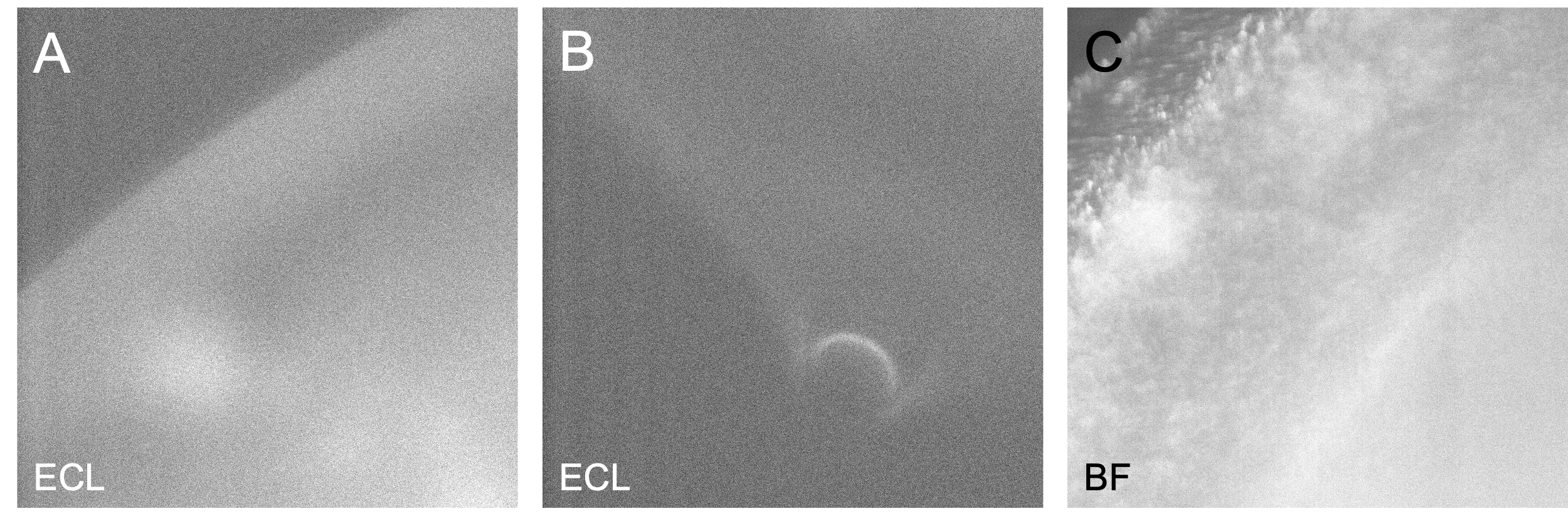
**

C

**Figure S10. Increased magnification on a platinum macroelectrode using a 40x objective. (A)** ECL micrograph displaying the triple phase boundary. **(B)** ECL micrograph displaying a bubble that was formed during the same trial (A) was taken. **(C)** Brightfield micrograph displaying the organic droplet near the triple phase boundary. Heterogeneities are the result of undissolved BPO crystals, which is a common occurrence at the aqueous|organic interface.

These trials were conducted to examine if there is surface blocking occurring on the electrode surface to account for the surprisingly low emission from using a platinum macroelectrode. Given that there were no clear abnormalities on the surface of the electrode, we have concluded that the electrode is likely being blocked by platinum hydrides rather than bubbles or the electroprecipitation product.

**
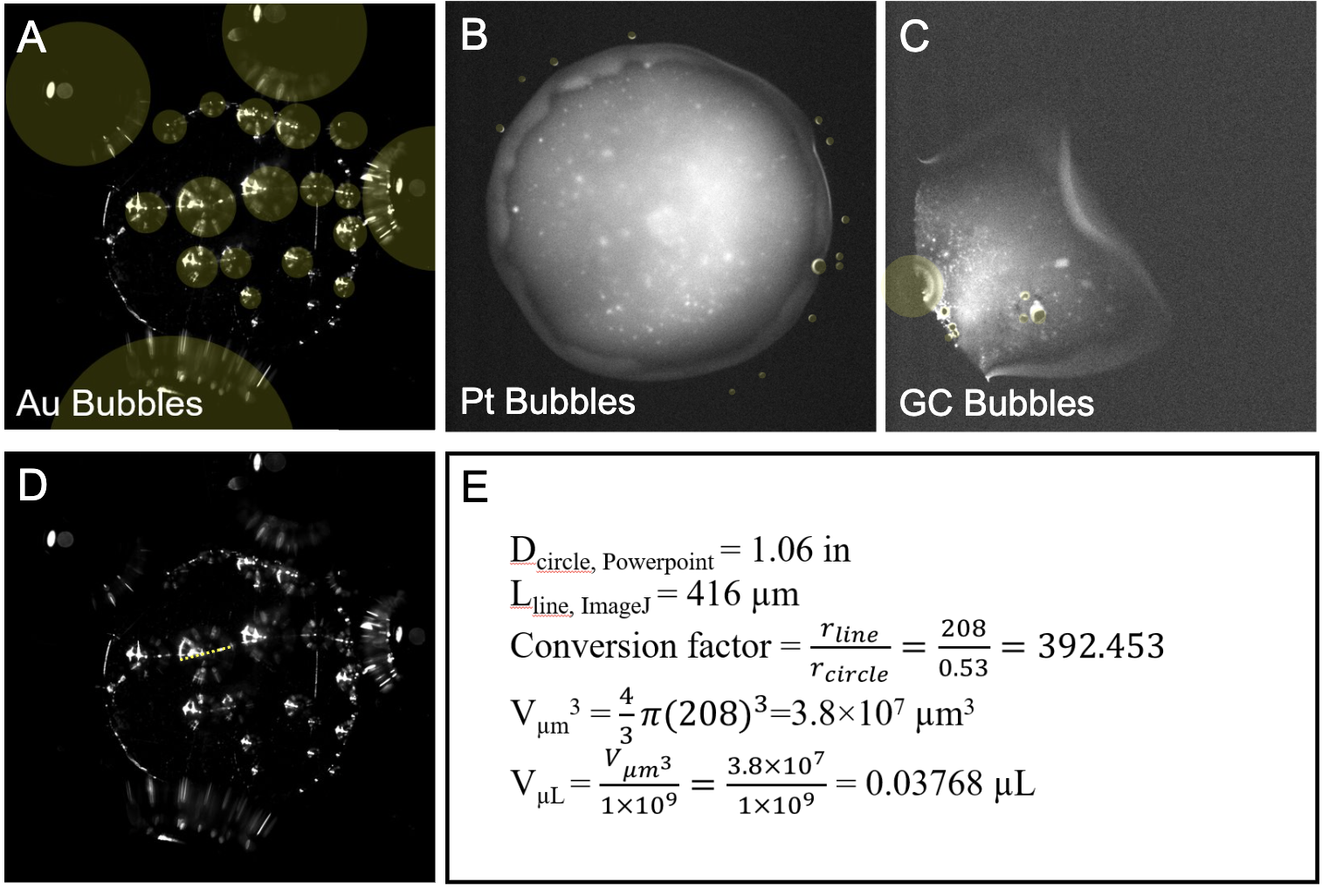
**

**Figure S11. Overlaid micrographs and example calculation of an individual bubble’s volume. (A)** Micrograph of gold macroelectrode bubble production, with circles drawn to outline the bubbles using Microsoft Powerpoint. **(B)** Micrograph of platinum macroelectrode bubble production, again with circles drawn to highlight the bubbles. **(C)** Micrograph of glassy carbon macroelectrode bubble production, again with circles drawn to highlight the bubbles. **(D)** Example of a line drawn in ImageJ to capture the actual diameter of a bubble. **(E)** Calculations involved to estimate the volume of each bubble. Detailed explanations of each step are as follows:

A circle is drawn over each bubble in the micrograph and the diameter of the circle according to Microsoft PowerPoint is recorded (in our case it was in inches), denoted by D_circle,PowerPoint_. The original micrograph is then uploaded to ImageJ where a straight line is drawn across the same bubble and measured in microns, denoted by L_line,ImageJ_. We then found a pseudo-stoichiometric conversion factor allowing for a conversion between the radius of the circle in PowerPoint and the “radius” of the line drawn in ImageJ. Note that this method is for ease of calculation and was done because some bubbles were only partially lit. Due to this, the true diameter without assistance or assumption is not entirely visible, but enough was visible to draw a circle of similar size. This conversion factor method was verified to work with all bubbles in the same micrograph but must be recalculated for a different micrograph. After the conversion factor is calculated and used to convert PowerPoint inches to ImageJ microns, the micron measurements were then used to calculate the volume of each bubble in cubic microns. After this, the volume was then converted to microliters, and all volumes in each micrograph were added together.


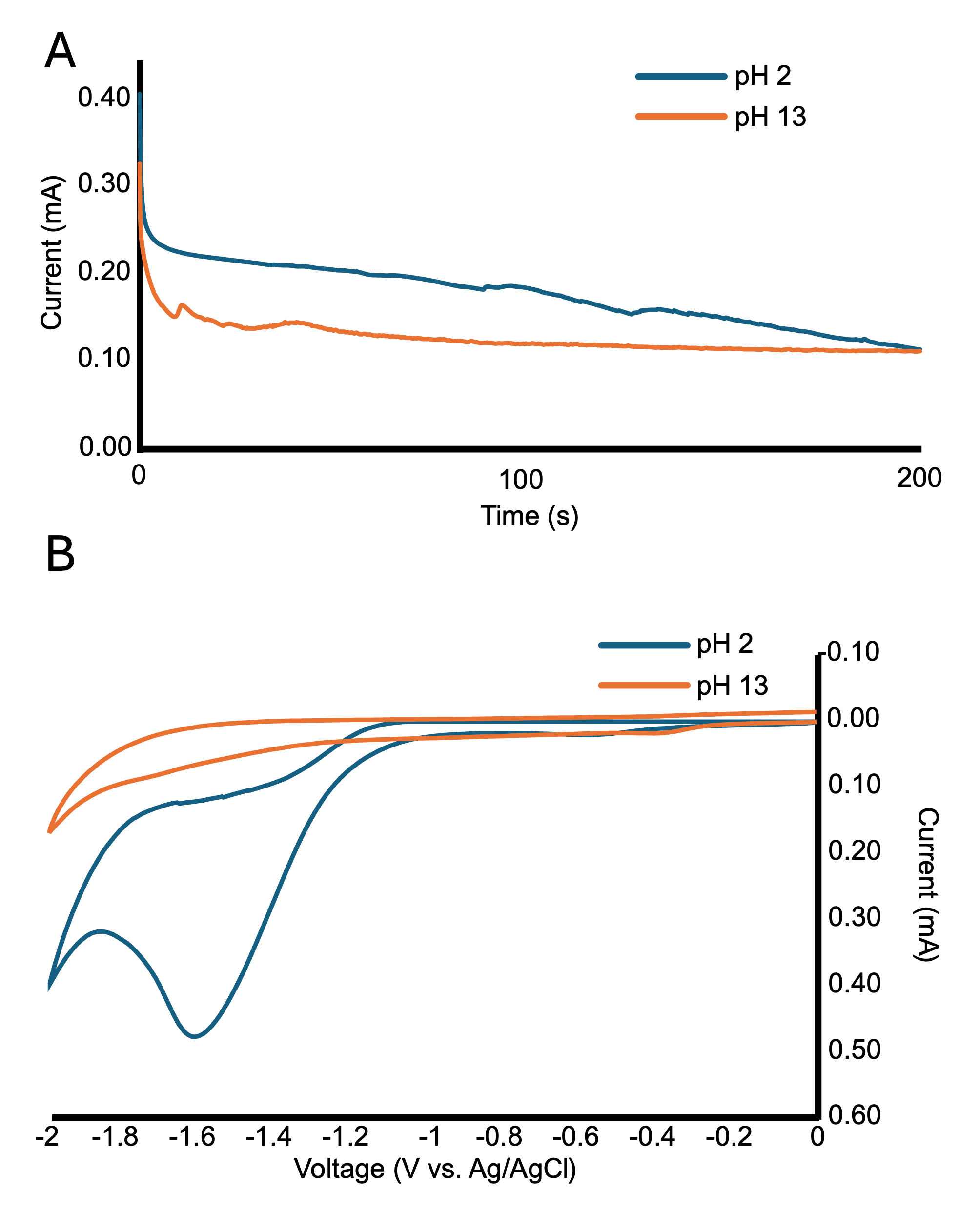


**Figure S12. Amperometric i-t curve and bulk Cyclic Voltammogram corresponding to Figure 4. (A).** Amperometric i-t curve at -1.8 V (vs. Ag/AgCl) in a hanging droplet configuration. The droplet phase consists of 10 mM [Ru(bpy)_3_]^2+^ and 100 mM KCl in water, which had been spiked with several drops of 1 M HCl to lower the pH to approximately 2, or several drops of 1M NaOH to raise the pH to 13. The bulk phase contains 50 mM BPO and 100 mM TBAP in 1,2-DCE. Phases were emulsified in a 1:25 ratio (200 µL and 5 mL, respectively). **(B)** Cyclic voltammetry of acidic (blue, pH 2) and basic (orange pH 13) bulk 100 mM KCl in water. The peak present in the redox is likely due to electrode blocking due to bubble nucleation. Scan rate was 50 mVs^-1^ with a quiet time of 10 s. The working electrode was an inlaid disk macroelectrode, the reference was a (1 M KCl) Ag/AgCl electrode, and the counter electrode was a glassy carbon rod.

**SI Movie Descriptions**

**SI Movie 1.** Corresponds to main text Figure 1D. The droplet phase contains 10 mM [Ru(bpy)_3_]^2+^ and 100 mM KCl in H_2_O. The bulk phase contains 50 mM BPO and 100 mM TBAP in 1,2-DCE.

**SI Movie 2.** Corresponds to main text Figure 2D. The droplet phase contains 1 mM [Ru(bpy)_3_]^2+^, 50 mM BPO, and 100 mM TBAP in 1,2-DCE. The bulk phase contains 10 mM [Ru(bpy)_3_]^2+^ and 100 mM KCl in H_2_O.

**SI Movie 3.** Corresponds to main text Figure 3B, row labelled “Au”. The droplet phase contains 1 mM [Ru(bpy)_3_]^2+^, 50 mM BPO, and 100 mM TBAP in 1,2-DCE. The bulk phase contains 10 mM [Ru(bpy)_3_]^2+^ and 100 mM KCl in H_2_O. A gold macroelectrode was used.

**SI Movie 4.** Corresponds to main text Figure 3B, row labelled “Pt”. The droplet phase contains 1 mM [Ru(bpy)_3_]^2+^, 50 mM BPO, and 100 mM TBAP in 1,2-DCE. The bulk phase contains 10 mM [Ru(bpy)_3_]^2+^ and 100 mM KCl in H_2_O. A platinum macroelectrode was used.

**SI Movie 5.** Corresponds to main text Figure 3B, row labelled “GC”. The droplet phase contains 1 mM [Ru(bpy)_3_]^2+^, 50 mM BPO, and 100 mM TBAP in 1,2-DCE. The bulk phase contains 10 mM [Ru(bpy)_3_]^2+^ and 100 mM KCl in H_2_O. A glassy carbon macroelectrode was used.

**SI Movie 6.** Corresponds to main text Figure 4A. The droplet phase contains 10 mM [Ru(bpy)_3_]^2+^, and 100 mM KCl in H_2_O, and has been spiked with enough 1 M HCl to reduce the pH to about 2. The bulk phase contains 50 mM BPO and 100 mM TBAP in 1,2-DCE.

**SI Movie 7.** Corresponds to main text Figure 4B. The droplet phase contains 10 mM [Ru(bpy)_3_]^2+^, and 100 mM KCl in H_2_O, and has been spiked with enough 1 M NaOH to increase the pH to about 13. The bulk phase contains 50 mM BPO and 100 mM TBAP in 1,2-DCE.
